# Home screening for bacteriuria in children with spina bifida and clean intermittent catheterization

**DOI:** 10.1186/1471-2334-12-264

**Published:** 2012-10-20

**Authors:** Bas SHJ Zegers, Cuno CSPM Uiterwaal, Carla C Verpoorten, Myleen MH Christiaens, Jan JLL Kimpen, Catharine CCE de Jong-de Vos van Steenwijk, Jan JD van Gool

**Affiliations:** 1 Department of Pediatrics, Máxima Medical Center Veldhoven, The Netherlands and Wilhelmina Children’s Hospital, University Medical Center Utrecht, Utrecht, The Netherlands; 2 Julius Center for Health Sciences and Primary Care, University Medical Center Utrecht, Utrecht, The Netherlands; 3 Department of Pediatrics, University Hospital Leuven, Leuven, Belgium; 4 Wilhelmina Children’s Hospital, University Medical Center Utrecht, Utrecht, The Netherlands; 5 Institute for Medical Informatics, Biometry and Epidemiology, University Hospital Essen, Essen, Germany; 6 Máxima Medical Center, Department of Pediatrics, PO Box 7777, 5500, MB, Veldhoven, The Netherlands

**Keywords:** Bacteriuria, Clean intermittent catheterization, Dip slide, Home testing, Leukocyte esterase test, Spina bifda

## Abstract

**Background:**

Significant bacteriuria (SBU) and urinary tract infections (UTIs) are common in patients with spina bifida and neuropathic detrusor sphincter dysfunction. Laboratory agar plated culture is the gold standard to establish SBU. It has the disadvantage of diagnostic and subsequent therapeutic delay. Leukocyte esterase tests (LETs) and dip slides proved to be useful in the general populations to exclude SBU and UTI. The aim of this study was to evaluate the reliability of LET and dip slide in children with spina bifida without symptoms of UTI. The reliability in children with asymptomatic SBU was not studied before.

**Methods:**

In one hundred and twelve children with spina bifida on clean intermittent catheterization LETs and dip slides were compared with laboratory cultures. Both tests and agar plated cultures were performed on catheterized urine samples. The hypothesis was that the home tests are as accurate as laboratory cultures.

**Results:**

A SBU was found in 45 (40%) of the 112 laboratory cultures. A negative LET excluded SBU (negative predictive value 96%), while a positive LET had a positive predictive value of 72%. The false positive rate was 28%. Dip slide determination of bacterial growth had no added value, other than serving as transport medium.

**Conclusions:**

In spina bifida children, leukocyte esterase testing can be used to exclude significant bacteriuria at home, while dip slide tests have no added value to diagnose or exclude significant bacteriuria.

## Background

Clean intermittent catheterization (CIC) and antibiotic prophylaxis have reduced the incidence of parenchymal kidney damage in children with spina bifida [1-3]. In these patients, the main objective of any urinary diagnostic test is to detect or exclude urinary tract infections (UTIs) to prevent under- and over-treatment. The diagnosis of UTI is made on clinical symptoms, leukocyturia and significant bacteriuria (SBU). Several simple tests to detect a UTI, such as dip slides and leukocyte esterase tests (LET) were studied extensively. Two recent meta-analyses showed that a LET had a negative predictive value (NPV) of 90%, with a positive predictive value (PPV) of 60% [4,5]. A UTI therefore has to be confirmed with a urine culture [4], which takes at least three days, and treatment is postponed. Dip slides with two or three culture media were tested to diagnose SBU in primary care [6-9]. As PPV was poor, it was concluded that the use of dip slide urine cultures should only be used to exclude SBU*.*

The aim of the present study was to evaluate the reliability of the LET and dip slide in children with spina bifida. Children with clinical symptoms of UTI participated in a parallel study, and were not included in this study [10]. Only children with asymptomatic SBU were included. We also assessed whether general patient characteristics, such as sex, age and use of prophylactic antibiotics can predict asymptomatic SBU. The hypothesis was that LET and Uricult® Duo dip slide are as accurate as laboratory cultures in determining significant bacteriuria.

## Patients and methods

One hundred and twelve patients with spina bifida on CIC known at the Gasthuisberg University Hospital Leuven, Belgium participated in the study. Patients catheterized themselves or were catheterized by their parents or primary care takers for a fresh urine sample at the quarterly control visit. Patients who had completed treatment for UTI less than 4 weeks before the visit to the clinic, or who had febrile episodes immediately preceding the visit, or a clinical suspicion for a UTI at the visit were excluded. A leukocyte esterase test (LET, Combur-2® test strip, Roche, Switzerland) was performed on the urine sample, regarded “positive” in every range of discoloration. The sample was also inoculated onto a dip slide (Uricult® Duo, Orion Diagnostics, Finland), which contains an aselective cystine-lactose-electrolyte deficient (CLED) agar for Gram-positive bacteria and *enterobacteriaceae*, and MacConkey agar for non-glucose fermenting Gram-negative rods. The dip slide was incubated in a bottle warmer (Philips® SBC 215/00, Philips SA, Belgium) at 36.3 ± 2.5 °C, as measured over 48 hours with a calibrated Dickson® SK 180 temperature logger (Dickson Corporation, Addison, USA). After 24 hours in the bottle warmer, the dip slide was evaluated for colony forming units by a trained research nurse, and the result was reported as ‘no growth’ or ‘growth’ (visible colonies). The same urine sample was also sent for ‘gold standard’ agar plated culture. SBU was defined as a colony count of ≥10^4^ per milliliter of one single species in a catheterized sample. Of patients with multiple samples, only the first was used for analysis. To establish whether general patient characteristics could discriminate SBU from no SBU, logistic regression was used with gold standard outcome (positive / negative) as dependent variable and age and sex as independent variables. This model was then extended with prophylaxis (model 2), LET testing (model 3), and dip slide testing (model 4). Model results are expressed as odds ratios (95% confidence intervals, and p-values). Discriminative capacity for these four models was evaluated using areas under the Receiver Operator Characteristic (ROC) curves (AUC). This study is approved by the ethics committee from the Leuven University Hospital, and performed after parental or guardian consent.

## Results

**Figure 1:**
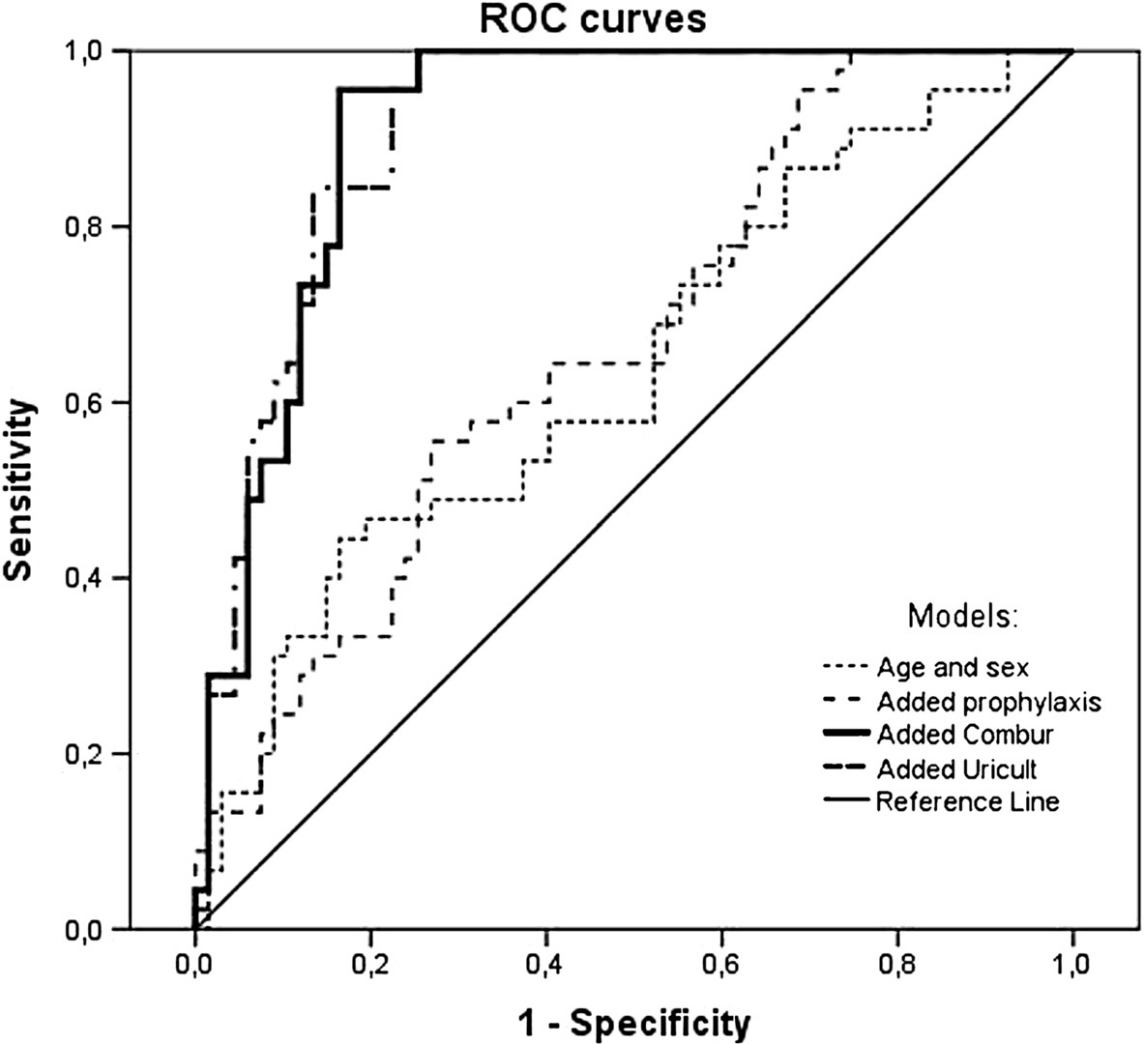
ROC curves of age, sex, Combur LET and Uricult dipslide

Of the 112 asymptomatic patients, 45 had a positive agar plated culture, hence the prior probability for SBU was 40%, which is consistent with previous studies. The patients had an age range of 0 to 35 years (median 13.0, long-term spina bifida follow-up patients over 18 years of age are included). Fifty (45%) were boys and 61 (68%) were on antibiotic prophylaxis. Table 1 shows the results of four consecutive models predicting the gold standard culture outcome. The LET had the strongest discriminative power, while dip slide testing did not add significantly. In Figure 1, the discriminative capacity of the models is shown graphically as ROC curves for age and sex (AUC = 0.64, p = 0.01); age, sex and prophylaxis (AUC = 0.76, p = 0.003); age, sex, prophylaxis and LET (AUC = 0.91, p < 0.0001) and age, sex, prophylaxis, LET and dip slide (AUC = 0.91, p < 0.0001). As this study addressed the role of LET and dip slide to rule out SBU in spina bifida patients without complaints of UTI, we proceeded with these tests only, as shown in Table 2. Given an a-priori chance of SBU of 40%, a positive LET had a PPV of 72%, while a negative LET substantially decreased the chance of a SBU to 4% (NPV = 96%). Dip slide testing had a similar PPV (73% versus 72% for LET) but substantially lower NPV (78% versus 96% for LET). Combining LET with dip slide improved neither PPV (positive LET 72% versus both LET and dip slide positive 74%) nor NPV (negative LET 96% versus both LET and dip slide negative 98%). Pathogens found were Escherichae coli (N=26), Klebsiella pneumonia (4), Streptococcus species (4), Enterococcus species (3), Proteus mirabilis (3), Pseudomonas aeruginosa (2), Serratia marcescens (1), Staphylococcus aureus (1) and Providencia rettgeri (1). In three of the 45 positive cultures (one Streptococcus, one Staphylococcus and one Enterococcus species) the LET was negative, resulting in 93.3% sensitivity. There were 16 false positive LETs in 67 negative cultures, resulting in 76% specificity.

**Table 1:** Determinants of significant bacteriuria

**Model**	**Odds ratio**		**95% CI**	**p-value**
1 Sex (male vs female)	1.9		0.9 - 4.2	0.10
Age (yrs)	1.06		1.01 - 1.12	0.03
2 Sex (male vs female)	1.8		0.8 - 4.1	0.13
Age (yrs)	1.07		1.01 - 1.14	0.02
Prophylaxis (yes/no)	0.5		0.2 - 1.0	0.06
3 Sex (male vs female)	4.1		1.2 - 14.4	0.03
Age (yrs)	1.03		0.94 - 1.12	0.54
Prophylaxis (yes/no)	0.3		0.1 - 1.1	0.07
LET	101		18 - 583	<0.0001
4 Sex (male vs female)	3.8		1.07 - 13.4	0.04
Age (yrs)	1.03		0.95 - 1.13	0.46
Prophylaxis (yes/no)	0.3		0.1 - 1.1	0.07
LET	75		11 - 495	<0.0001
Dipslide	1.6		0.5 - 5.4	0.46

LET = leukocyte esterase test.

**Table 2:** Predictive value of (combinations of) Leukocyte Esterase Test and dipslide for significant bacteriuria

**Tests**		**Gold standard**				
		**Positive**	**Negative**	**Total**	**PPV in % (95% CI)**	**NPV in % (95% CI)**
LET	Positive	43	17	60	72 (50 – 83)	
	Negative	2	50	52		96 (85 – 99)
Dipslide	Positive	29	11	40	73 (56 – 85)	
	Negative	16	56	72		78 (66 – 87)
Combi	Both positive	28	10	38	74 (57 – 87)	
	Not both positive	17	57	74		77 (66 – 86)
Combi	Not both negative	44	18	62	71 (58 – 82)	
	Both negative	1	49	50		98 (89 – 99)

## Discussion

In this study of 112 spina bifida patients on clean intermittent catheterization, a negative LET excludes SBU in a home setting with a NPV of 96%. A negative dip slide alone was not effective to rule out SBU, and a negative LET together with a negative dip slide did not improve NPV. Both a positive LET and dip slide had a false positive rate of more than 20 percent compared to laboratory cultures, and cannot be used to diagnose SBU.

### Leukocyte esterase test

Our results are consistent with other studies and meta-analyses, performed in the general pediatric populations [4,5,11-15]. Anderson et al. studied the LET in children with neurogenic bladders, combined with nitrite test, with comparable results [16]. Adversely, in a similar study population, Liptak et al. found a lower NPV (83%) [17]. A significantly lower NPV for the LET (68%) was also seen in adults with spinal cord injury, which could be attributed to their lower threshold to diagnose SBU, with 10^2^ colony forming units per milliliter of catheterized urine. In this study, the threshold was 10^4^ cfu/ml [18].

In this study, boys had a significantly higher risk of SBU than girls. In a study by Seki et al., girls with myelodysplasia were more likely to get colonized with bacteria [19]. Age did not influence the risk for SBU in our population, in accordance with previous studies [20]. Prophylactic antibiotics tended to reduce the risk of SBU, as was shown in previous studies both in the general population [21-23] and in patients with spina bifida [22,24,25]. Compared to the LET however, age, sex and the use of antibiotic prophylaxis are less reliable to predict SBU in children with spina bifida.

### Dip slides

In this study, a negative dip slide with a NPV of 78% and a false negative rate of 22% could not rule out SBU. With a PPV of 73%, and a false positive rate of 27%, SBU cannot be diagnosed with a dip slide. In a recent study in 200 children with UTI symptoms and a positive LET, Uricult® Trio dip slides incubated in a laboratory incubator were compared with colony counts on blood agar plates. The sensitivity of 68%, and a false negative rate of 29% was comparable to this study [26]. Two mayor pitfalls were found: the small pin-point colonies of some *Enterococci* and most *Streptococci* on the CLED medium were mistaken for no growth, and transparent *E. coli* colonies are almost invisible. The untrained eye can be aided by the European Urinalysis Guidelines [27]. Inspection of the dip slide with a 12× magnifying glass, and comparing the incubated media with those of an unused dip slide. When growth of *E coli*, *Enterococci*, and *Streptococci* on the 14 false negative Uricult® Duo dip slides was identified in this study, the false negatives would have decreased from 27% to 14%.

This study included only asymptomatic patients, and although the bacteriuria is significant, this has no clinical consequences such as therapeutic antibiotic administration. Compared to asymptomatic SBU, in clinical UTI leukocyturia is obligatory, most likely increasing both NPV and PPV of the LET, emphasizing the value of the LET. A further study to evaluate the reliability of the dip slide in children with spina bifida and clinical symptoms of UTI is recommended.

## Conclusion

In home testing of spina bifida children on clean intermittent catheterization, leukocyte esterase testing can be used to exclude significant bacteriuria. Both leukocyte esterase test and dip slide are not sensitive enough to predict significant bacterial growth, and a agar plated culture should therefore be performed when either test is positive. Other than serving as transport medium, dip slide testing has no added to diagnose or exclude significant bacteriuria.

## Abbreviations

CI: Confidence interval; CIC: Clean intermittent catheterization; CLED: Cystine-lactose-electrolyte deficient; LET: Leukocyte esterase test; NPV: Negative predictive value; PPV: Positive predictive value; SBU: Significant bacteriuria; UTI: Urinary tract infection.

## Competing interests

The authors declare that they have no competing interests.

## Authors’ contributions

BZ, CU, CJ and JG participated in conception and design of this study. BZ, CV and MC performed the study, included participants and acquired the data. BZ, CU and CJ analyzed and interpreted the data. BZ wrote the original manuscript, CU, CV, MC, CJ, JK and JG revised the article and approved the final manuscript. All authors read and approved the final manuscript.

## Pre-publication history

The pre-publication history for this paper can be accessed here:

http://www.biomedcentral.com/1471-2334/12/264/prepub

## References

[B1] Dik P Klijn AJ van Gool JD van de Jong-de Vos Steenwijk CC De Jong TP Early start to therapy preserves kidney function in spina bifida patients Eur Urol 2006 49 0302–2838; 5 908 913 16458416 10.1016/j.eururo.2005.12.056

[B2] De Jong TP Chrzan R Klijn AJ Dik P Treatment of the neurogenic bladder in spina bifida Pediatr Nephrol 2008 23 0931–041; 6 889 896 18350321 10.1007/s00467-008-0780-7 PMC2335291

[B3] Ozel SK Dokumcu Z Akyildiz C Avanoglu A Ulman I Factors affecting renal scar development in children with spina bifida Urol Int 2007 79 1423–0399; 2 133 136 17851282 10.1159/000106326

[B4] Deville WL Yzermans JC van Duijn NP Bezemer PD van der Windt DA Bouter LM The urine dipstick test useful to rule out infections. A meta-analysis of the accuracy BMC Urol 2004 4 1471–2490 4 15175113 10.1186/1471-2490-4-4 PMC434513

[B5] St John JA Boyd JC Lowes AJ Price CP The use of urinary dipstick tests to exclude urinary tract infection: a systematic review of the literature Am J Clin Pathol 2006 126 0002–9173; 3 428 436 16880133 10.1309/C69RW1BT7E4QAFPV

[B6] Aspevall O Forsum U Kjerstadius T Hallander H Evaluation of two methods for improving quality of diagnosis of bacteriuria by culture in primary healthcare Scand J Clin Lab Invest 2000 60 0036–5513; 5 387 393 11003258 10.1080/003655100750019297

[B7] Duerden BI Moyes A Comparison of laboratory methods in the diagnosis of urinary tract infection J Clin Pathol 1976 29 0021–9746; 4 286 291 777041 10.1136/jcp.29.4.286 PMC476048

[B8] Aspevall O Kjerstadius T Lindberg L Hallander H Performance of Uricult Trio assessed by a comparison method and external control panels in primary healthcare Scand J Clin Lab Invest 2000 60 0036–5513; 5 381 386 11003257 10.1080/003655100750019288

[B9] Winkens R Nelissen-Arets H Stobberingh E Validity of the urine dipslide under daily practice conditions Fam Pract 2003 20 0263–2136; 4 410 412 12876111 10.1093/fampra/cmg412

[B10] Zegers B Uiterwaal C Kimpen J van Gool J de Jong T Winkler-Seinstra P Houterman S Verpoorten C De Jong-de Vos Van Steenwijk C Antibiotic prophylaxis for urinary tract infections in children with spina bifida on intermittent catheterization J Urol 2011 186 6 2365 2370 10.1016/j.juro.2011.07.108 22019031

[B11] Little P Turner S Rumsby K Warner G Moore M Lowes JA Smith H Hawke C Turner D Leydon GM Arscott A Mullee M Dipsticks and diagnostic algorithms in urinary tract infection: development and validation, randomised trial, economic analysis, observational cohort and qualitative study Health Technol Assess 2009 13 1366–5278; 19 1 iii-xi 19364448 10.3310/hta13190

[B12] Lunn A Holden S Boswell T Watson AR Automated microscopy, dipsticks and the diagnosis of urinary tract infection Arch Dis Child 2010 95 193 197 1468–2044; 0003–9888; 3 10.1136/adc.2009.166835 19815534

[B13] Mori R Yonemoto N Fitzgerald A Tullus K Verrier-Jones K Lakhanpaul M Diagnostic performance of urine dipstick testing in children with suspected UTI: a systematic review of relationship with age and comparison with microscopy Acta Paediatr 2010 99 1651–2227; 0803–5253; 4 581 584 20055779 10.1111/j.1651-2227.2009.01644.x

[B14] Whiting P Westwood M Bojke L Palmer S Richardson G Cooper J Watt I Glanville J Sculpher M Kleijnen J Clinical effectiveness and cost-effectiveness of tests for the diagnosis and investigation of urinary tract infection in children: a systematic review and economic model Health Technol Assess 2006 10 1366–5278; 36 1 iii-xiii 17014747 10.3310/hta10360

[B15] Williams GJ Macaskill P Chan SF Turner RM Hodson E Craig JC Absolute and relative accuracy of rapid urine tests for urinary tract infection in children: a meta-analysis Lancet Infect Dis 2010 10 1474–4457; 1473–3099; 4 240 250 20334847 10.1016/S1473-3099(10)70031-1

[B16] Anderson JD Chambers GK Johnson HW Application of a leukocyte and nitrite urine test strip to the management of children with neurogenic bladder Diagn Microbiol Infect Dis 1993 17 0732–8893; 1 29 33 8359003 10.1016/0732-8893(93)90066-g

[B17] Liptak GS Campbell J Stewart R Hulbert WC Jr Screening for urinary tract infection in children with neurogenic bladders Am J Phys Med Rehabil 1993 72 0894–9115; 3 122 126 8512672 10.1097/00002060-199306000-00003

[B18] Hoffman JM Wadhwani R Kelly E Dixit B Cardenas DD Nitrite and leukocyte dipstick testing for urinary tract infection in individuals with spinal cord injury J Spinal Cord Med 2004 27 1079–0268; 1079–0268; 2 128 132 15162883 10.1080/10790268.2004.11753743

[B19] Seki N Masuda K Kinukawa N Senoh K Naito S Risk factors for febrile urinary tract infection in children with myelodysplasia treated by clean intermittent catheterization Int J Urol 2004 11 0919–8172; 11 973 977 15509200 10.1111/j.1442-2042.2004.00943.x

[B20] Bakke A Vollset SE Risk factors for bacteriuria and clinical urinary tract infection in patients treated with clean intermittent catheterization J Urol 1993 149 0022–5347; 3 527 531 8437255 10.1016/s0022-5347(17)36136-0

[B21] Conway PH Cnaan A Zaoutis T Henry BV Grundmeier RW Keren R Recurrent urinary tract infections in children: risk factors and association with prophylactic antimicrobials JAMA 2007 298 1538–3598; 2 179 186 17622599 10.1001/jama.298.2.179

[B22] Craig JC Simpson JM Williams GJ Lowe A Reynolds GJ McTaggart SJ Hodson EM Carapetis JR Cranswick NE Smith G Irwig LM Caldwell PH Hamilton S Roy LP Antibiotic prophylaxis and recurrent urinary tract infection in children N Engl J Med 2009 361 1533–4406; 1533–4406; 18 1748 1759 19864673 10.1056/NEJMoa0902295

[B23] Dai B Liu Y Jia J Mei C Long-term antibiotics for the prevention of recurrent urinary tract infection in children: a systematic review and meta-analysis Arch Dis Child 2010 95 1468–2044; 0003–9888; 7 499 508 20457696 10.1136/adc.2009.173112

[B24] Clarke SA Samuel M Boddy SA Are prophylactic antibiotics necessary with clean intermittent catheterization? A randomized controlled trial J Pediatr Surg 2005 40 1531–5037; 3 568 571 15793737 10.1016/j.jpedsurg.2004.11.027

[B25] Morton SC Shekelle PG Adams JL Bennett C Dobkin BH Montgomerie J Vickrey BG Antimicrobial prophylaxis for urinary tract infection in persons with spinal cord dysfunction Arch Phys Med Rehabil 2002 83 0003–9993; 1 129 138 11782843 10.1053/apmr.2002.26605

[B26] Anacleto FE Resontoc LP Padilla GH Bedside diagnosis of outpatient childhood urinary tract infection using three-media dipslide culture test Pediatr Nephrol 2009 24 1432–198; 8 1539 1543 19495802 10.1007/s00467-009-1217-7

[B27] European urinalysis guidelines Scand J Clin Lab Invest Suppl 2000 231 0085–591 1 86 12647764

